# Protocol for correlation of histological risk assessment/scoring system with a depth of invasion in oral squamous cell carcinoma

**DOI:** 10.12688/f1000research.134757.2

**Published:** 2024-05-31

**Authors:** Archana Sonone, Alka Hande, Aayushi Pakhale, Madhuri Gawande, Swati Patil

**Affiliations:** 1 Department of Oral & Maxillofacial Pathology and MicrobiologySharad Pawar Dental College & Hospital, Datta Meghe Institute of Higher Education, Wardha, Maharashtra, 442004, India

**Keywords:** Oral squamous cell carcinoma, Brandwein Gensler Criteria, Depth of Invasion

## Abstract

**Introduction:**

The commonest type of cancer in the head and neck region is oral squamous cell carcinoma (OSCC) due to its high rates of occurrence and mortality. The early diagnosis of oral cancer gives better prognosis. Brandwein-Gensler criteria predict the early stage of OSCC cases with a high risk of locoregional recurrence.

**Objectives:**

To correlate Brandwein-Gensler criteria and depth of invasion of OSCC with three-year survival.

**Methodology:**

In the study, This study will include 80 random histopathologically-diagnosed cases of OSCC. hematoxylin-eosin (HE)-stained section slides will be used to evaluate, Brandwein and Gensler criteria by three histopathologists in a blinded manner. The depth of invasion assessment will be done from the basement-membrane (BM), in regions where the BM has been lost, as well as from an illustrative line connecting the BM from the neighbouring epithelium to the point of deepest tumour invasion in the connective-tissue stroma with the help of a research microscope (Leica-DMLB2) in resected tissue specimens of OSCC cases.

**Expected results:**

The present study will find the correlation between Brandwein-Gensler criteria and depth of invasion in OSCC in order to evaluate the locoregional recurrence in OSCC cases. In high-risk OSCC cases, there may be an increased depth of invasion in resected tissues.

**Conclusions:**

We hypothesized that the correlation between Brandwein-Gensler criteria and depth of invasion can be used as an independent predictor for locoregional recurrence in OSCC.

## Introduction

Oral squamous cell carcinoma (OSCC) is the most widely occurring cancer in the head-neck region. ^1^ In 2018, OSCC claimed around 177,000 global fatalities. ^2^ Despite of the advanced treatment modalities and therapeutic approaches, the overall survival rate in OSCC did not rise by more than 50% during a five-year period. ^3^ The clinical diagnosis of OSCC is confirmed by histopathology. In the adjunct therapy such as chemotherapy radiotherapy, histopathological report of OSCC is important. Due to this, many studies emphasize on the histopathological parameters of resected oral cancer tissues in the pathology reports. ^4^ Several authors have proposed various grading systems for OSCC. The first grading system developed by Broder (1920), which is still advised by the WHO ^5^^,^^6^ is likely the most well-known prognostication method using a subjective evaluation of significant histopathological features like differentiation degree of tumor cells, cellular pleomorphism, and mitotic activity, and is grouped into WDSCC (well differentiated), MDSCC (moderately differentiated) and PDSCC (poorly differentiated). Although MDSCC comprises up to 90% of oral cancers, this approach, while widely identified and used, still has poor discriminatory value because in MDSCC pathologically there is a poor cellular differentiation than the WDSCC. ^6^^,^^7^ Annoreth *et al.* gave another classification which emphasise on the association between the tumour and adjacent tissue. ^8^ This included aspects like the leukocyte invasion and the degree of pattern and stage of invasion. A method of invasive-tumour-front grading (IFG) comprising five histopathological characteristics involving lymphocyte-host-response (LHR) was later devised by Bryne *et al*. ^9^ Because of the small sample sizes, varied locations of the tumour, and examination of various types of specimens, these models have not proved successful. A risk assessment model put forth in 2005 by Brandwein-Gensler and colleagues was said to have better prognostic value than previously mentioned systems. ^10^ It includes the combined assessment of three important histopathological parameters: PNI (perineural-invasion), LHR (lymphocyte-host-response), and WPOI (worst-pattern of invasion). Scoring is done for all three parameters individually and then the sum of the three variables is computed. The Brandwein-Gensler (BG) system showed a significant correlation with the patient’s locoregional recurrence and overall survival, especially in low-stage oral cancers. More aggressive characteristics receive weighted point values in the model. ^11^ Adjuvant radiotherapy may be beneficial in cases of high-risk, low-stage oral cancers, even when the margins are satisfactory. The TNM classification for OSCC (eight edition, 2017), emphasizes on depth-of-tumour invasion (DOI) along with largest diameter of the tumour to determine the T-stage ^12^ and signifies a shift in the paradigm in Oral Pathology. When determining the specific course of treatment for a particular case, a combined evaluation of clinical staging and histological grading may be a more reliable method. ^1^ The main goal of our study is to determine the association of DOI with the histological risk assessment/scoring system in order to assess the combined effects of these factors on OSCC pathological staging and locoregional recurrence in three-year survival rate.

## Methods

### Trial design

This study will be retrospective, using archival tissue from OSCC cases.

### Study setting

This cross-sectional study will be carried out at Sharad Pawar Dental College and Hospital (SPDC&H), Datta Meghe Institute of Higher Education, Sawangi-Meghe, Wardha in the Oral Pathology and Microbiology Department. This study will include 80 random histopathologically-diagnosed cases of OSCC. Computer-generated randomization technique was used. The staging will be carried out on the basis of the American Joint Committee on Cancer (AJCC) eighth edition criteria. ^13^ Cases with a previous head-neck cancer history, pre-surgical radiation therapy, chemotherapy or any surgery (apart from biopsy) and recurrent or distant disease will be excluded. Patient data comprising of age and sex of the patient, site of the lesion, clinical appearance of the tumor, habits including tobacco and alcohol use and status of lymph node will be obtained.

### Intervention

Staining of the archival slides and new slides (where needed) will be done with Heamtoxyline and eosin stain. Worst-pattern of invasion WPOI, Lymphocyte-host-response LHR, and Perineural-invasion PNI will be assessed by three histopathologists in a blinded manner. Evaluation will be done according to the Risk Model mentioned below. Three groups will be made depending on the sum of the score of each case:

Group 1 – low-risk cases = total score 0

Group 2 – moderate-risk cases = 1 or 2

Group 3 – high risk-cases = 3 or more.

The DOI assessment will be done from the basement-membrane (BM), in regions where the BM has been lost, as well as from an illustrative line connecting the BM from the neighbouring epithelium to the point of deepest tumour invasion in the connective-tissue stroma with the help of a research microscope (Leica-DMLB2) with standard software (Leica-Q-win). The DOI will be measured and recorded.

### Risk assessment system derived from Brandwein-Gensler et al.

**WPOI:**

**WPOI-1:** Broad-pushing borders – **Score 0**

**WPOI-2:** Broad-pushing fingers – **Score 0**

**WPOI-3:** Large tumour cells islands (with more than fifteen tumour cells in each island) – **Score 0**

**WPOI-4:** Small islands of tumour (less than fifteen tumour cells in each island) – **Score +1**

**WPOI-5:** Satellites of tumour cells, at ≥1mm distance from the major tumour – **Score +3**

**Lymphocytic-host-response (LHR):**

**LHR-1 (strong):** Dense rim of lymphoid infiltrate surrounding the tumour at the advancing edge/invasive front – **Score 0**

**LHR-2 (intermediate):** patches of lymphoid infiltrate in few regions – **Score +1**

**LHR-3 (weak):** Presence of very little or no lymphoid infiltrate, no patches seen – **Score +3**

**Perineural-invasion:**

**No invasion – Score 0**

**Small nerves:** Tumour cells surrounding small nerves – **Score +1**

**Large nerves:** Tumour cells surrounding nerves, ≥1 mm in diameter – **Score +3**

### Outcomes

The present study will find the correlation between Brandwein-Gensler criteria and DOI in OSCC. In the high-risk category, there may be increased DOI in resected tissue of OSCC.

### Sample size

Considering the prevalence of OSCC as 70.7% in the outpatient department of the Oral Pathology and Microbiology department, using the single proportion formula, sample size is calculated by applying the formula:

$$n\geq Z21-\alpha /2\;*p*\left(1-p\right)/d\;2$$

Where,

Z21−α/2 - The significance level at 5%

*i.e.* 95 % confidence interval = 1.96

p = Sample showing positive CD44 expression focally in small group cells in the basal layer of epithelium = 70.7% = 0.707

E=Error of margin=10%=0.10

$n=1.96^{2}\times 0.707\times \left(1-0.707\right)/0.10^{2}$

n = 80

### Dissemination

The results will be published in an indexed journal.

### Study status

The study has not started yet.

## Discussion

Incidence of OSCC in Indian men (11.28%) ranks first while it ranks fifth in women (4.3%). ^1^^,^^2^ The clinical diagnosis is always confirmed by the histopathology. There are numerous histopathological grading systems mentioned in the literature for OSCC. Among them, the BG risk predictive model gives the best results regarding the loco-regional recurrence of OSCC. ^4^

Arun Chaturvedi evaluated the BG risk predictive model in surgically treated OSCC patients in North India. The author studied 149 patients with histologically diagnosed OSCC. The patients were divided into three groups: low, moderate, and high risk according to the BG criteria. Out of 149 patients, 17 patients showed locoregional recurrences (11.4%). Most of these 17 patients belonged to the high-risk category of the BG risk model. The authors hypothesised that the BG risk model is predictive of locoregional recurrences for OSCC undergoing primary surgery and it can be used to model for recognition of recurrences and prevention at early stages of the disease. ^14^

Rhayany de Castro Ribeiro Lindenblatt studied 53 cases of OSCC in which they evaluated four grading systems (Malignancy Grading of the Deep Invasive Margins, Multiparameter Grading System, Histologic Risk Assessment and the World Health Organization grading system). They concluded that the Histologic Risk Assessment scoring system established the best results for locoregional recurrence and survival prediction in OSCC patients. ^15^

Naomi Rahman evaluated the BG risk model criteria in OSCC patients to assess disease progression and survival of patients. They studied 134, OSCC cases, in which they compared two AJCC criteria, *i.e.* seventh and eighth. In the latest eighth criteria they included DOI according to which they staged the tumor. Due to this, 30 cases of OSCC previously classified into the T1 and T2 stage according to the seventh edition were upstaged to T3 stage according to the eighth AJCC criteria. Three individual histopathologists scored BG risk model criteria and DOI of the same samples. They concluded that the BG is the most effective parameter for grading tumors and locoregional recurrence of patients. ^16^

The staging of OSCC, which is based on the TNM system, has been used for several years to estimate both the clinical response to therapy and survival outcomes. In the eighth AJCC edition, DOI is included as a new histopathological parameter for the assessment of tumor grade, The DOI is measured from the basement membrane to invasive the front of the tumor. The stage and grade of the tumor are major contributing factors to prognosis and treatment selection for OSCC. ^17^ As the BG risk model gives the locoregional recurrence status of OSCC, assessment and corelation of BG model and depth of tumor give the best results in predicting three-year survival in locoregional recurrence of OSCC patients.

## Conclusions

For OSCC, the BG risk model comprises effective criteria to determine locoregional recurrence. Therefore, it can be used in early-stage OSCC for identification and prevention of the recurrences. The correlation between BG and DOI in resected OSCC tissues will give better results about the locoregional recurrence and survival rate of OSCC.

### Ethical considerations

Ethical approval was received from Datta Meghe Institute of Higher Education and Research, Sawangi, Wardha, Maharashtra India (IEC reference number- DMIHER (DU)/IEC/2023/578).

## Data Availability

No data are associated with this article. Zenodo: STROBE checklist for “Protocol for correlation of histological risk assessment/scoring system with a depth of invasion in oral squamous cell carcinoma”, https://doi.org/10.5281/zenodo.7895574 . ^18^ Data are available under the terms of the Creative Commons Attribution 4.0 International license (CC-BY 4.0).
