# Supplementary material for: Protocol for correlation of histological risk assessment/scoring system with a depth of invasion in oral squamous cell carcinoma
Source: F1000Res. 2024 May 31;12:1326. Originally published 2023 Oct 12. [Version 2] doi: 10.12688/f1000research.134757.2 (PMC11384190; doi:10.12688/f1000research.134757.2)
Supplement: Peer review history

# Reviewer report: Reviewer response for version 2

**DOI:** 10.5256/f1000research.167199.r314720

**Published:** 2024-09-09

**Authors:** Jose A. Hardillo

**Affiliations:** 1 Erasmus MC Cancer Institute, Rotterdam, The Netherlands

I have read the revised form of this protocol for correlation of histological risk assessment/scoring system with a depth of invasion in oral squamous cells carcinoma well. I believe that this will be a good and relevant study. I do not have major concerns regarding the protocol except that the authors should clearly define that they are going to include only T1N0 and T2N0 tumors.

Is the study design appropriate for the research question?

Yes

Is the rationale for, and objectives of, the study clearly described?

Yes

Are sufficient details of the methods provided to allow replication by others?

Yes

Are the datasets clearly presented in a useable and accessible format?

Yes

Reviewer Expertise:

head and neck tumor surgery, oligometastasis

I confirm that I have read this submission and believe that I have an appropriate level of expertise to confirm that it is of an acceptable scientific standard.

# Reviewer report: Reviewer response for version 2

**DOI:** 10.5256/f1000research.167199.r285383

**Published:** 2024-07-08

**Authors:** Alessandro Polizzi

**Affiliations:** 1 University of Catania, Catania, Italy

In their revised version the authors successfully addressed my comments. The protocol may be accepted after the following minor correction:

 _ ABSTRACT, Correct the errors in the sentences: “In the study, This study will include 80 random histopathologically-diagnosed cases of OSCC. hematoxylin-eosin…”.

Is the study design appropriate for the research question?

Yes

Is the rationale for, and objectives of, the study clearly described?

Yes

Are sufficient details of the methods provided to allow replication by others?

Partly

Are the datasets clearly presented in a useable and accessible format?

Not applicable

Reviewer Expertise:

Oral pathology and medicine; periodontology, orthodontics, oral surgery.

I confirm that I have read this submission and believe that I have an appropriate level of expertise to confirm that it is of an acceptable scientific standard.

# Reviewer report: Reviewer response for version 1

**DOI:** 10.5256/f1000research.147837.r261195

**Published:** 2024-04-12

**Authors:** Alessandro Polizzi

**Affiliations:** 1 University of Catania, Catania, Italy

In the manuscript entitled “Protocol for correlation of histological risk assessment/scoring system with a depth of invasion in oral squamous cell carcinoma” the authors aimed to present a protocol to correlate Brandwein-Gensler criteria and depth of invasion of OSCC with three-year survival.

 Here are reported some suggestions to improve the quality of the manuscript:

**ABSTRACT:**

- Please, give more details about the methodology and study design in the abstract.

**INTRODUCTION:**

- The authors reported that “Although MDSCC comprises up to 90% of oral cancers, this approach, while widely identified and used, still has poor discriminatory value”. Please, better explain why it has poor discriminatory value.
- “The Brandwein-Gensler (BG) system showed a significant correlation with the patient’s locoregional recurrence and overall survival, especially in low-stage oral cancers”. Please, give more details about the results you found in the literature about the strengths of this correlation related to recurrence and overall survival.
-  “The TNM classification for OSCC (eigtth edition…”. Please correct the error.

**METHODS:**

-  Please better explain if this study will be a retrospective or a cross-sectional study.
- The authors cited the AJCC eighth edition criteria. Please explain the acronym and add a reference.
- Please, give more details about the randomized selection of histopathologically-diagnosed cases of OSCC.
- “Staining of the archival slides and new slides (where needed) will be done with HE. WPOI, LHR, and PNI will be assessed by three histopathologists in a blinded manner”. Please specify all the acronyms the first time they have been cited.

Is the study design appropriate for the research question?

Yes

Is the rationale for, and objectives of, the study clearly described?

Yes

Are sufficient details of the methods provided to allow replication by others?

Partly

Are the datasets clearly presented in a useable and accessible format?

Not applicable

Reviewer Expertise:

Oral pathology and medicine; periodontology, orthodontics, oral surgery.

I confirm that I have read this submission and believe that I have an appropriate level of expertise to confirm that it is of an acceptable scientific standard, however I have significant reservations, as outlined above.

# Response

**Published:** 2024-05-28

**Authors:** Dr. Archana Sonone

**Affiliations:** Oral Pathology & Microbiology, Sharad Pawar Dental Collge and Hospital sawangi meghe wardha, Wardha, Maharashtra, India

Dear Reviewer, 

 Thank you for your response , I have done all the corrections which were mentioned in the comment. 

 The Brandwein-Gensler (BG) system showed a significant correlation with the patient’s locoregional recurrence and overall survival, especially in low-stage oral cancers”. Please, give more details about the results you found in the literature about the strengths of this correlation related to recurrence and overall survival. meaning of this sentence- this point elaborated in the discussion section .

 Thank you regards,

 Dr. Archana Sonone
